# Dobrava Hantavirus Infection Complicated by Panhypopituitarism, Istanbul, Turkey, 2010

**DOI:** 10.3201/eid1807.111746

**Published:** 2012-07

**Authors:** Nevin Sarıgüzel, Jörg Hofmann, Alper Tunga Canpolat, Ali Türk, Jakob Ettinger, Deniz Atmaca, Işın Akyar, Serap Yücel, Ender Arıkan, Yavuz Uyar, Dilek Y. Çağlayık, Ayşe Sesin Kocagöz, Ayşin Kaya, Detlev H. Kruger

**Affiliations:** Acıbadem Hospital, Istanbul, Turkey (N. Sarıgüzel, A.T. Canpolat, D. Atmaca, Serap Yücel, Ender Arıkan);; Charité University Medicine, Berlin, Germany (J. Hofmann, J. Ettinger, D.H. Kruger);; Labor Berlin Charité-Vivantes GmbH, Berlin (J. Hofmann, J. Ettinger, D. H. Kruger);; Acıbadem University, Istanbul (A.Türk, I. Akyar, A. S. Kocagöz);; Refik Saydam National Public Health Agency, Ankara, Turkey (Y. Uyar, D. Y. Çağlayık);; and University of Geneva, Geneva, Switzerland (A. Kaya)

**Keywords:** Hantavirus, Dobrava-Belgrade virus, renal failure, respiratory distress, panhypopituitarism, viruses

## Abstract

We identified Dobrava-Belgrade virus infection in Turkey (from a strain related to hantavirus strains from nearby countries) in a patient who had severe symptoms leading to panhypopituitarism, but no known risk for hantavirus. Our findings emphasize the need for increased awareness of hantaviruses in the region and assessment of symptomatic persons without known risk factors for infection.

Hemorrhagic fever with renal syndrome (HFRS) is caused by infection with hantaviruses. Most patients with HFRS recover completely, but acute and chronic complications may develop. HFRS patients with severe lung involvement resembling hantavirus cardiopulmonary syndrome have been described (*1*). In addition, pituitary hemorrhage, followed by hypopituitarism, is a possible complication of HFRS. Involvement of the pituitary gland has been observed in some patients infected with Puumala virus, a hantavirus commonly found in western and central Europe (*2**–**4*).

We report on a patient who experienced shock, pulmonary failure, and panhypopituitarism as complications of HFRS. By testing for neutralizing antibodies and by amplification and molecular characterization of virus samples, we identified the causative pathogen as a strain of Dobrava-Belgrade virus (DOBV) that is closely related to a hantavirus strain typically carried by the yellow-necked field mouse (*Apodemus flavicollis*), strain DOBV-Af.

## The Case-Patient

A 34-year-old man with fever (38°C), tender cervical lymph nodes, and symptoms of pharyngeal infection was admitted to a hospital in Istanbul, Turkey on February 3, 2010. His blood pressure was 120/70 mm Hg and heart rate was 96 beats/min. Laboratory findings included thrombocytopenia (63,000 platelets/mm^3^ [reference 130–450×10^3^ platelets/mm^3^]) and mild elevation of transaminase levels (alanine transaminase 95 IU/L [reference 10–40 IU/L]; Aspartate aminotransferase 99 IU/L [reference 15–40 IU/L]) (Table 1). Results of urinalysis, chest radiograph, and ultrasound of the abdomen were normal. Diarrhea developed during the first day of hospitalization, and conjunctival suffusion was observed on ocular examination. Ciprofloxacin was given to treat suspected salmonellosis at a dosage of 400 mg every 12 hours.

**Table 1:** Hematologic and biochemical parameters of DOBV hantavirus patient, Istanbul, Turkey, 2010*

Laboratory test (reference range)	Day of hospitalization								
	0	1	2	3	4	5	15	20	71
Leukocytes, × 10^3^ cells/mm^3^ (4.5–11)	5.8	9.9	15.2	27.6	24	25	11.3	4.7	7.7
Hemoglobin, g/dL (11.7–15.5)	16	16.5	18	16	13.3	10.7	8.2	8.9	12.1
Hematocrit, % (36–46)	46.7	48	51.8	45.2	38.3	31.2	24.6	27	36.6
Platelets, × 10^3^/mm^3^ (130–450)	63	19	20	13	37	55	180	171	254
ALT, U/L (10–40)	95	92	58	67	157	539	79	60	20
AST, U/L (15–40)	99	117	–†	–	497	2234	106	84	25
BUN, mg/dL (4.67–23.3)	–	18	23	30	41	41	129	74	19
Creatinine, mg/dL (0.7–1.2)	–	0.8	1.8	2.4	3.3	3.1	7.3	6.4	1.92
aPTT, s (4–40)	–	39	43	–	32.5	37.1	30.8	–	–
PT, s (10–14 s)	–	13.3	11.6	–	17.5	20	14.1	–	–
Ferritin, ng/mL (15–200)	–	5,304	–	–	14,036	–	–	–	–
TSH, U/mL (0.4–4.2)	–	1.71	–	–	–	–	–	2.83	0.92
Free thyroxin, pmol/L (10.3–23.2)	–	–	–	–	–	–	–	0.18	14.3
Cortisol, μg/dL (6.2–19.4)	–	–	–	–	–	45.0	–	0.98	4.7
ACTH, pg/mL (0–46)	–	–	–	–	–	3.02	–	6.80	–
ADH, pg/mL (0.8–4.5)	–	–	–	–	–	–	–	2.2	–
Total testosterone, ng/mL (2.8–8.0)	–	–	–	–	–	–	–	<0.025	1.9
LH, mIU/mL (1.7–8.6)	–	–	–	–	–	–	–	<0.1	–
FSH, mIU/mL (1.5–12.4)	–	–	–	–	–	–	–	0.66	–
Prolactin, mIU/mL (4.6–21.4)	–	–	–	–	–	–	–	10.05	–
Somatomedin-C/IGF-1, ng/mL (115–307)	–	–	–	–	–	–	–	<25	–

*DOBV, Dobrava-Belgrade virus; ALT, alanine transaminase; AST, aspartate aminotransferase; BUN, blood urea nitrogen; aPTT, activated partial thromboplastin time; PT, prothrombin time; TSH, thyroid stimulating hormone; ACTH, adrenocorticotropic hormone; ADH, antidiuretic hormone; LH, luteinizing hormone; FSH, follicle-stimulating hormone; IGF, insulin-like growth factor. †Test not performed.

On day 2 of hospitalization, the patient became oliguric and hypotensive. He had persisting fever. His platelet count decreased to 20,000/mm^3^, and his serum creatinine level increased to 1.8 mg/dL (reference 0.7–1.2 mg/dL). Urinalysis results showed microscopic hematuria and proteinuria. Results of a transthoracic echocardiogram showed heart chambers of normal size, a left ventricular ejection fraction of 60%, and moderate pericardial effusion. The patient was transferred to the intensive care unit. Broad-spectrum antimicrobial drugs were initiated as treatment for suspected sepsis, and platelet and albumin transfusions were given as supportive therapy.

On day 3 of hospitalization, the patient experienced tachycardia, petechial lesions appeared on his extremities, and his platelet count dropped to 13,000/mm^3^. Results of a bone marrow aspiration showed proliferation of histiocytes and prominent hemophagocytosis. Intravenous methylprednisolone therapy (100 mg/day) was started. Septic shock developed in the patient, and inotropic therapy was initiated. On the same day, acute respiratory distress syndrome developed, and assisted ventilation was started. Results of a thoracic computerized tomographic scan showed focal infiltration on the upper zone of the left lung and minimal pleural effusion on the right lung. Results of abdominal computerized tomographic scan revealed ascites and multiple mesenteric lymphadenomegalies. On the basis of these clinical and laboratory findings, hantavirus infection was suspected. Blood samples were obtained for hantavirus testing, and oral ribavirin was added at an initial dose of 30 mg/kg, followed by 15 mg/kg every 6 hours for 4 days, then 7.5 mg/kg for 6 days. On the same day, hemofiltration was started and continued for 6 consecutive days. The patient’s urinary output gradually increased, pulmonary symptoms regressed, and his ventilation tube was removed on day 12 of hospitalization. The patient stayed in the intensive care unit for 18 days and was then transferred to a standard care unit.

No microbial growth was observed in the cultures of samples taken during the first days of hospitalization. Serum samples were tested for antibodies against a panel of pathogens, including *Salmonella typhi, S. paratyphi* A and B, *Brucella* spp., HIV, Crimean-Congo hemorrhagic fever virus, Epstein-Barr virus, cytomegalovirus, parvovirus B19, adenovirus, dengue virus, *Rickettsia* spp., *Leptospira* spp., *Treponema pallidum*, and hepatitis viruses A, B, C, and E. Results of these serologic tests were negative or inconspicuous. Results of PCR analysis conducted for leptospirosis, respiratory viruses, and cytomegalovirus were below the detection limit of the assays.

**Figure 1:**
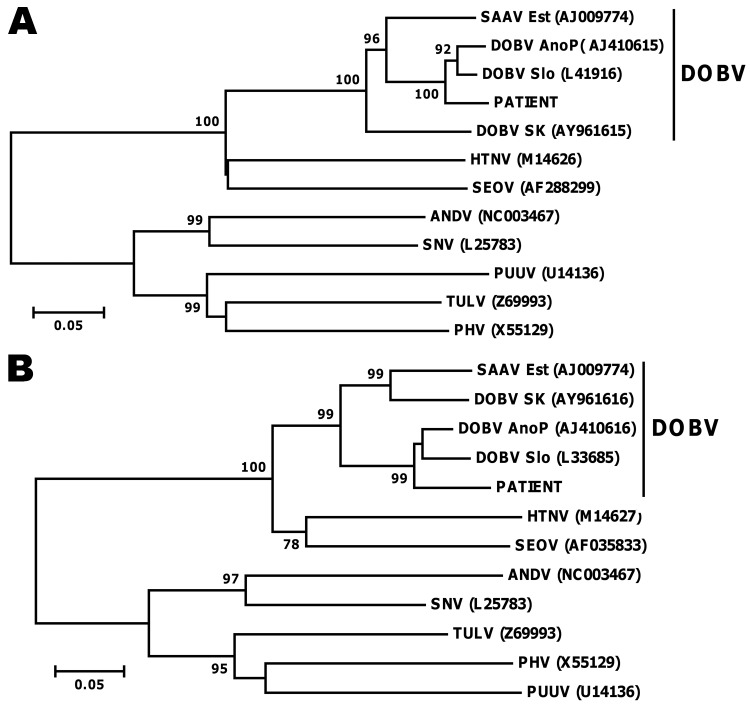
Molecular phylogenetic analysis of small (S) and medium (M) gene segments. Consensus neighbor-joining phylogenetic tree (Tamura-Nei 93 evolutionary model) of hantavirus strains was constructed as described (*9*) based on partial sequences of the S (panel A) and M segment (panel B). Bootstrap values >70%, calculated from 10,000 replicates, are shown at the tree branches. Sequences taken from GenBank are indicated by their accession numbers. SAAV Est, Saaremaa virus from Estonia; DOBV AnoP, Dobrava-Belgrade virus (lineage DOBV-Af) from Greece; DOBV Slo, Dobrava-Belgrade virus (lineage DOBV-Af) from Slovenia; DOB SK, Dobrava-Belgrade virus (lineage DOBV-Aa) from Slovakia; HTNV, Hantaan virus; SEOV, Seoul virus; ANDV, Andes virus; SNV, Sin nombre virus; PUUV, Puumala virus; TULV, Tula virus; PHV, Prospect Hill virus. Scale bars indicate an evolutionary distance of 0.1 substitutions per position.

Serologic testing for hantavirus was performed by using the *recom*Line Bunyavirus IgG/IgM immunoassay (Mikrogen, Neuried, Munich, Germany); results showed strong reactivity for IgG and IgM antibodies, indicating an acute hantavirus infection. The blot data provided evidence of an infection with DOBV; to confirm the results, we performed serotyping by using focus reduction neutralization tests (Table 2). The results confirmed a DOBV infection most likely caused by a strain of DOBV-Af. Reverse transcription PCR results for hantaviral RNA were positive for the first acute-phase blood sample. Subsequent nucleotide sequence determination of parts of the 3 genomic segments and molecular phylogenetic analysis of these small, medium (Figure 1), and large gene sequences (data not shown) showed that the isolate was most closely related to DOBV-Af.

**Table 2:** Characterization of DOBV hantavirus in a patient’s serum by focus reduction neutralization tests, Istanbul, Turkey, 2010*

Day of hospitalization	Focus reduction neutralization test endpoint titer			
	Puumala hantavirus	Hantaan virus	DOBV-Aa (strain SK)	DOBV-Af (strain Slo)
5	<1:40	<1:40	1:640	1:1,280
26	<1:40	<1:40	1:160	1:320

*DOBV, Dobrava-Belgrade virus; Aa, *Apodemus agrarius*; Af, *Apodemus flavicollis;* SK, strain Slovakia; Slo, strain Slovenia.

**Figure 2:**
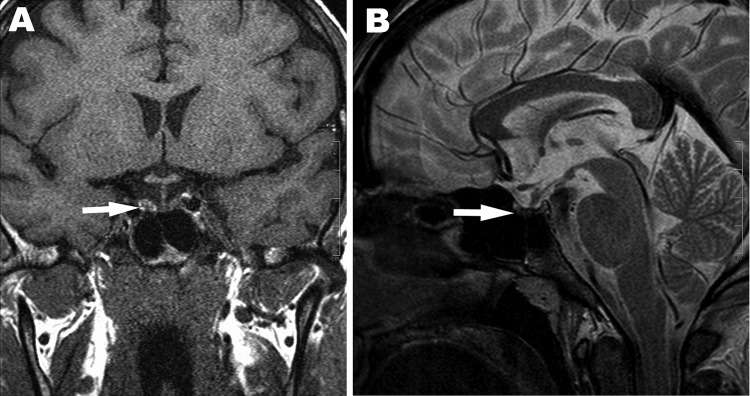
Magnetic resonance images showing hemorrhage of the pituitary gland and pituitary atrophy as indicated by arrows. A) T1-weighted coronal image shows high signal intensity on the right side of the adenohypophysis consistent with hemorrhage. B) T2-weighted sagittal image shows decreased pituitary gland height and heterogenous low signal intensity of the central adenohypophysis due to hemorrhagic infarction.

On day 19 of hospitalization, the patient displayed generalized weakness. The results of the basal levels of hormonal studies are shown in Table 1. On day 20 of hospitalization, panhypopituitarism was diagnosed. Hormone replacement therapy using hydrocortisone, thyroxin, and testosterone was immediately initiated. On day 22 of hospitalization, magnetic resonance imaging showed pituitary hemorrhage and pituitary atrophy (Figure 2). After the administration of hormone replacement therapy, symptoms improved markedly. The patient was discharged on day 33 of hospitalization. At the final outpatient followup visit, the patient had normal urine output and his serum creatinine level was 1.5 mg/dL. His pulmonary examination results and chest radiograph findings were normal, but he continued to require hormonal replacement therapy at the 16th month of followup care.

## Conclusions

The general symptoms and results of the serologic tests and molecular analyses for this patient were consistent with a hantavirus infection. After the acute phase of the illness, characterized by renal and pulmonary failure, panhypopituitarism developed in the patient. Pituitary hemorrhage and atrophy were observed on the magnetic resonance image. Case reports have documented that pituitary hemorrhage followed by panhypopituitarism may complicate HFRS (*2**–**4*), and Puumala virus was reported as the causative agent in most of these cases. In addition, hormonal deficiencies have been shown to be common features of Puumala virus infections, and chronic hormonal deficits develop in some patients (*5*). Our findings demonstrate that development of hypopituitarism can also be associated with infection by DOBV. Since the patient does not belong to any groups that have high exposure to hantaviruses (such as farmers, forest workers, and military recruits) and did not report any travel in hantavirus-endemic areas, the source of infection remains unclear.

In contrast to what is known about hantavirus presence in other Balkan states, little is known about the distribution, diversity, or host range of hantaviruses in Turkey (*6*). In 2009, a hantavirus outbreak occurred in Turkey; 3 of the 5 infected persons died (*7*). Serologic assays have been used to detect DOBV infection in patients in Turkey, but focus reduction neutralization tests have not been used for serotyping. DOBV RNA was found only in 1 urine sample from a hantavirus-infected patient from Turkey; however, sequence data were not provided (*8*). Prevalence studies of rodents collected in Turkey revealed 6% seropositivity to Puumala virus in *Microtus* voles and no appearance of hantavirus antibodies in the *Apodemus* species of rodents (*9*). Focus reduction neutralization testing and virus sequencing confirmed that the patient in our study was infected with DOBV-Af. This strain is associated with *A. flavicollis* field mice and yet has not been reported in animals in Turkey (see *10**,**11* for the molecular classification of *Apodemus* spp.–associated DOBV virus lineages).

This case should alert physicians that hantavirus infection should be considered in the differential diagnosis of patients who have high fever and thrombocytopenia, including those without known risk factors for hantavirus exposure. Since severe hormonal deficiencies are life-threatening, neuroendocrinologic complications should be taken into account and the endocrine status should be investigated to prevent panhypopituitarism even after recovery from HFRS.
